# Comparative study of Revogene GBS LB assay and GeneXpert GBS LB assay for the detection of group B *Streptococcus* in prenatal screening samples

**DOI:** 10.1186/s12879-019-4756-y

**Published:** 2020-01-14

**Authors:** Tsokyi Choera, Brittney Jung-Hynes, Derrick J. Chen

**Affiliations:** 1 Department of Medical Microbiology & Immunology, University of Wisconsin- Madison, 1505 Adams Dr, Menlo Park, CA 94025 USA; 2 Dept. Pathology and Laboratory Medicine, University of Wisconsin School of Medicine and Public Health, 3170 MFCB, 1685 Highland Ave, Madison, WI 53705 USA

**Keywords:** *Streptococcus agalactiae*, Group B *Streptococcus*, Nucleic acid amplification test, Molecular test, Perinatal prevention, GBS comparison

## Abstract

**Background:**

Group B Streptococcal (GBS) infections in the United States are a leading cause of meningitis and sepsis in newborns. The CDC therefore recommends GBS screening for all pregnant women at 35–37 weeks of gestation and administration of intrapartum prophylaxis (in those that tested positive) as an effective means of controlling disease transmission. Several FDA approved molecular diagnostic tests are available for rapid and accurate detection of GBS in antepartum women.

**Method:**

In this study, we report a clinical comparison of the Xpert GBS LB assay and a novel FDA-cleared test, Revogene GBS LB assay. A total of 250 vaginal-rectal swabs from women undergoing prenatal screening were submitted to the University of Wisconsin’s clinical microbiology laboratory for GBS testing.

**Results:**

We found 96.8% of samples were concordant between the two tests, while 3.2% were discordant with a positive percent agreement of 98.0% and a negative percent agreement of 96.5% between the Revogene GBS LB assay and the GeneXpert GBS LB assay.

**Conclusion:**

Overall, we report that both assays perform well for the detection of GBS colonization in pregnant women.

## Background

*Streptococcus agalactiae,* often referred to as Group B *Streptococcus* (GBS), is a gram-positive bacterium found in the rectum and vagina of approximately 25% of pregnant women [1]. While GBS is an asymptomatic colonizer of most healthy adults, it can cause severe infections in neonates, including sepsis, pneumonia, and meningitis [2, 3]. GBS early onset disease (EOD) are infections that occur in the first week of life and can be extremely dangerous to the newborn; EOD occurred in 3 per 1000 live births and was associated with a high mortality rate prior to the 1990s [4]. Due to the high neonatal mortality rate caused by GBS infections, the Centers for Disease Control (CDC) implemented a universal guideline in 1996 which recommended screening of all pregnant women at 35–37 weeks of gestation and administration of intrapartum prophylaxis in pregnant women that tested positive [1, 5].

Despite these guidelines, infection with GBS remains a leading cause of morbidity in neonates born in the United States and therefore the implementation of more rapid and sensitive screening techniques for GBS detection may further reduce transmission of GBS infection intrapartum [1, 6, 7]. The current gold standard for GBS detection is enrichment of the primary specimen, a vaginal-rectal swab, followed by subculture onto a blood agar plate with phenotypic characterization [1]. This gold standard method has a long turnaround time, and sensitivity is only 54–87% [8, 9]. Furthermore, bacterial culture requires an experienced technician to further identify and test characteristics of GBS such as agglutination and beta hemolysis [1].

Although culture remains the gold standard in GBS diagnostics, the 2010 CDC revision for GBS testing allows for nucleic acid amplification tests (NAAT) such as polymerase chain reaction (PCR) as an option for GBS testing [1]. A PCR method based on amplification of the CAMP factor encoding gene (*cfb*), a fragment that is present in nearly all GBS strains, was developed by Ke et al. [3] in 2000. Since then, several rapid and sensitive DNA probes and NAAT assays for GBS have been developed and approved or cleared by the Food and Drug Administration (FDA) [3, 10, 11]. In our study, we report a clinical comparison of two FDA cleared NAATs for the detection of GBS in antepartum women: Xpert GBS LB (Cepheid Inc., Sunnyvale, CA, USA) and a novel recently FDA-cleared test, Revogene GBS LB (Meridian Bioscience, Québec City, Canada). Both systems perform an automated nucleic acid extraction, real-time PCR amplification, and detection of the target nucleic acid sequences after LIM broth enrichment [12, 13].

## Methods

### Study specimens

A total of 250 vaginal-rectal swabs from pregnant women undergoing routine GBS screening were tested in the present study. The specimens were submitted to the University of Wisconsin’s clinical microbiology laboratory between December 2017 to February 2018. All swab specimens were first swirled in LIM broth (Todd Hewitt with Colistin and Nalidixic Acid) for 10 s. The swabs were then removed from broth and discarded. Inoculated LIM broths were held for 18–24 h in a CO_2_ incubator. Post-enrichment, each sample was clinically tested using the Xpert GBS LB assay, followed by testing with the Revogene GBS LB assay within 72 h. All results were recorded, and for discordant results, testing was repeated on each platform and cultures were performed. The same positive (ATCC 12386, *Streptococcus agalactiae*) and negative (ATCC 9809, *Streptococcus gallolyticus*) external controls were used in both assays. The limit of detection (LOD) for the Revogene GBS LB assay ranges from 200 to 375 CFU/mL depending on the ATCC GBS strains while the overall LOD for the GeneXpert GBS LB assay is 333 CFU/mL using ATCC or CDC GBS strains.

### Xpert GBS LB assay

All enriched samples were first tested on the GeneXpert Dx system using the Xpert GBS LB Assay according to the manufacturer’s instructions. Briefly, the LIM broth enriched samples were inverted 3 times and a sterile swab was immersed into the LIM broth enriched samples. This swab was immediately inserted into the sample chamber in the Xpert GBS LB cartridge and run on the GeneXpert Dx System.

### Revogene GBS LB assay

Following completion on the GeneXpert Dx system, the same LIM broth enriched specimens were tested on the Revogene system according to the manufacturer’s instructions. Briefly, the LIM broth enriched samples were vortexed for 15 s and diluted into the diluent buffer. The diluted samples were vortexed for another 15 s and loaded into Revogene GBS LB PIE and run on the Revogene.

### Bacterial culture testing

Reference culture testing was performed on all discordant samples in accordance with published CDC guidelines. Briefly, the LIM broth enriched samples were sub-cultured onto blood agar plates and incubated for 18 to 24 h at 37 °C in 5% CO_2_. The blood agar plates without any colonies observed at 24 h were re-incubated for an additional 24 h. If after 48 h no colonies formed, the culture was deemed negative. Any suspect colonies were identified via matrix-assisted laser desorption ionization time-of-flight mass spectrometry (MALDI-TOF MS). For MALDI-TOF MS-identified GBS isolates, 16S rRNA gene sequencing was conducted for confirmation.

### Time and cost

The hands-on setup time of each assay was timed and averaged over five individual runs. List price of each test and instrument were provided by vendors.

### Data analysis

Results from both GeneXpert and Revogene systems were compared. Concordant results between the assays were recorded, and no additional testing was performed. Discordant results between the initial tests were repeated on both instrument platforms and culture of the enriched specimens to check for GBS presence was also performed. Agreement between the two tests was calculated.

## Results

From the 250 total samples tested, 242 samples were concordant (96.8%), and eight samples were discrepant (3.2%) between the two molecular assays. Among the eight discrepant results, seven specimens tested negative by GeneXpert, but positive by Revogene and one tested positive by GeneXpert, but negative by Revogene with initial testing. All discrepant results were repeated by both molecular assays and set up for culture. Upon repeat testing, one discrepant sample that initially tested positive by GeneXpert, tested negative in the repeat testing. Upon repeat of the seven other discrepant samples that initially tested negative by GeneXpert, three samples tested positive and three samples tested negative upon repeat. Interestingly, one of the samples that had initially tested negative on the GeneXpert tested negative upon repeat on GeneXpert but tested positive on Revogene (initial and repeat) and had growth in culture. The strain isolated from that specimen was confirmed to be *Streptococcus agalactiae* by sequencing the entire 16S rRNA gene and by MALDI-TOF MS analysis. The results are summarized in Table 1.

**Table 1:** Comparison of results for the Xpert GBS LB Assay, the Revogene GBS LB Assay, and Culture

No. of specimens	Test Results				
	Xpert GBS LB assay	Revogene GBS LB assay	Repeat Xpert	Repeat Revogene	Culture^*^
192	Negative	Negative	N/A	N/A	N/A
50	Positive	Positive	N/A	N/A	N/A
3	Negative	Positive	Positive	Positive	Negative
2	Negative	Positive	Negative	Negative	Negative
1	Positive	Negative	Negative	Negative	Negative
1	Negative	Positive	Negative	Positive	Negative
1	Negative	Positive	Negative	Positive	Positive

^*^Culture was only performed for discrepant results between Xpert and Revogene

Positive, negative, and overall percent agreement (PPA, NPA, OPA) between the two assays were calculated, and the results are shown in Table 2. The PPA, NPA, and OPA between the Revogene and Xpert assays were 98.0% (50/51; 95% CI, 89.6–99.7%), 96.5% (192/199; 95% CI, 92.9–98.3%), and 96.8% (242/250; 95% CI, 93.8–98.4%), respectively.

**Table 2:** Performance characteristics of Revogene compared to Xpert GBS LB Assays

		Xpert		
		Positive	Negative	Total
Revogene	Positive	50	7	57
	Negative	1	192	193
	Total	51	199	250
		95% CI		
	Positive Percent Agreement	98.0%	89.6 to 99.7%	
	Negative Percent Agreement	96.5%	92.9 to 98.3%	
	Overall agreement	96.8%	93.8 to 98.4%	

Time and cost of each assay were also assessed as displayed in Table 3. The Revogene GBS LB test resulted in an average of 2.7 min to setup, and had a run time of 70 min, while the Xpert GBS LB assay required an average of 1.3 min to setup with a run time of 59 min per test. The list price for each test was $28 for Revogene and $30 for GeneXpert. The list price for the instruments when comparing equal numbers of testing modules, was $35,000 for Revogene and $110,000 for GeneXpert.

**Table 3:** Time and Cost Comparison between the Xpert GBS LB and the Revogene GBS LB Assays

Assay	Time (mins)		Cost ($)^a^	
	Setup Per Test	Run Time	Per Test^a^	Per Instrument^a^
Xpert GBS LB	1.3	59	30	110,000
Revogene GBS LB	2.7	70	28	35,000

^a^Cost is based on the list price provided by the vendors; each vendor works with individual labs to try to make these kits and instrument affordable within their budgets

**The test price is listed as per test; however, the tests are purchased as kits

## Discussion

The purpose of our study was to compare the newly FDA-cleared Revogene GBS LB assay to the GeneXpert GBS LB assay. While the small consumable and equipment size of the Revogene are an advantage in terms of space requirements and waste management, the GeneXpert allows for the flexibility of adding on more modules, has a more extensive test menu, and specimens are tested individually; the Revogene instrument requires batched testing of 1 to 8 samples per run [12, 13]. One shortcoming of the Revogene instrument during our testing was that we were not notified of any error when loading the pie onto the instrument until the run was complete; this caused error/invalid rates of 2.4% for the Revogene, while the GeneXpert had a 0% error/invalid rate. A major advantage of the Revogene was higher sensitivity, though based on repeat testing, there were more false positive results. It is noteworthy to highlight the single discrepant sample that resulted in GBS-negative GeneXpert results compared to GBS-positive Revogene and culture results (Table 1) [14].

Both the GeneXpert and the Revogene GBS assays’ primers and probes detect a target within or adjacent to the CAMP factor encoding gene (cfb) of GBS [4, 12, 13]. The cfb gene was believed to be present in almost all GBS isolates, yet recent findings have described cases of cfb-negative isolates that were missed by the GeneXpert system [15, 16]. The isolate that was recovered by culture from the discordant specimen was sent to Cepheid for further analysis, which revealed a rare deletion across the primer/probe region adjacent to the cfb gene [14].

## Conclusion

To summarize, we evaluated the performance and workflow characteristics of two commercially available platforms for GBS detection: the Revogene GBS assay and the GeneXpert GBS assay. 250 specimens were collected from women undergoing prenatal screening and our results showed that both assays had excellent agreement between each other (98.0% PPA, 96.5% NPA, 96.8% OPA). Both the setup and run times were slightly longer for the Revogene assay compared to that of the GeneXpert assay. The list price per test was less for the Revogene assay compared to that of GeneXpert assay. For the same number of testing modules, the Revogene instrument costs less than the GeneXpert Dx System based on list prices. Overall, both GBS assays and platforms perform well; however, there are specific differences listed in our study that laboratories should consider when deciding between the two.

## Data Availability

The datasets used and/or analyzed during the current study are available from the corresponding author on reasonable request.

## Abbreviations

CDC: Centers for Disease Control
*cfb*: CAMP factor encoding gene
EOD: Early onset disease
FDA: Food and Drug Administration
GBS: Group B *Streptococcus*
MALDI-TOF MS: Matrix-assisted laser desorption ionization time-of-flight mass spectrometry
NAAT: Nucleic acid amplification tests
